# Experiences of Older People and Social Inclusion in Relation to Smart “Age-Friendly” Cities: A Case Study of Chongqing, China

**DOI:** 10.3389/fpubh.2021.779913

**Published:** 2021-12-13

**Authors:** Manlin Li, Ryan Woolrych

**Affiliations:** School of Energy, Geoscience, Infrastructure and Society, The Urban Institute, Heriot-Watt University, Edinburgh, United Kingdom

**Keywords:** smart cities, urban communities, ageing, social inclusion, age friendly city, smart technology

## Abstract

Whilst cities can be sites of creativity, innovation, and change, they can also reproduce the conditions for the exclusion of vulnerable groups. Older people report experiencing specific barriers to accessing the city and are often excluded from the resources for ageing well. The smart city agenda has attempted to bring about technological change whilst also delivering improved quality of life for urban citizens. Smart technologies are a key element of the smart city and are viewed as having the potential to support the independence, autonomy, and well-being of older people. Yet, there has been little research exploring the role of the smart city in supporting the social inclusion of older people, nor any attempt to link this with key policy drivers on ageing e.g., age-friendly cities and communities. In response, the aim of this paper is to explore the experiences of older people living in a smart city in China and discuss how the smart city and age-friendly agenda can be brought together to support positive social outcomes for older people. The paper presents qualitative findings from a multi-methods approach, including semi-structured interviews, walking interviews and focus groups. A total of 64 older people participated in the research across three diverse neighbourhoods in the case study smart city of Chongqing, China. The findings identified opportunities in the development and deployment of smart city, including the potential for improved health and well-being and social connectedness. Yet in delivering on these benefits, a number of challenges were identified which may widen social inequalities, including inequities in access, issues of safety and security, and exclusion from the co-production of smart city policy and practise. The paper discusses the implications of the findings for future smart city policy and practise, specifically in delivering interventions that support older adults' social inclusion and the delivery of age-friendly cities and communities.

## Introduction

### Smart Cities: A Creative and Innovative Response to Ageing and Urbanisation?

Innovative communication technologies enable us to share and communicate at a distance; the growth of infrastructure networks and the spread of digitisation of information have helped to speed up urban evolution in every aspect of society (1). At the same time, research has emphasised the meaningful role of cities as agents of global change and key elements in driving social impact, often in unintended ways (2). Making the city smart is a relatively new urban development approach aimed at delivering sustainable urban environments through enhanced digital connectivity (3). Whilst there is no consensus on the definition of a smart city (4, 5), principles of sustainability, inclusivity, and integration are closely entwined. For example, the British Standards Institute (BSI) described a smart city as “*the effective integration of physical, digital, and human systems in the built environment to deliver a sustainable, prosperous, and inclusive future for its citizens*” (6).

Smart cities, through information transformation, networks of participation and social engagement introduce a new and potentially radical approach to urban development and current planning practise (7). Information Communication Technology (ICT) and the processing of “big data” have the potential to transform the way in which we live and communicate and thereby impact everyday lives (8, 9). The desire to enhance the quality, performance, and interactivity of urban services is a strong motivational factor, as is the drive to improve city infrastructure e.g., housing, transport, and outdoor spaces (10, 11). Yet, the deployment of the smart city agenda has also been heavily criticised, potentially opening up new forms of spatial inequalities as some groups remain digitally disconnected, and raising concerns about how smart cities can deliver inclusive outcomes for more vulnerable and hard to reach populations (12, 13).

Against the background of accelerated ageing and urbanisation, cities are seen as a hotbed for stimulating technological and social change (14). In delivering digital innovations, smart cities have the potential to respond to the twinned global trends of urbanisation and ageing which are shaping society and raising challenges and opportunities for how we design sustainable and equitable urban environments (15). In order to do so, our cities and urban policies have to provide innovative solutions to support an ageing population, providing essential interventions to meet the needs of older people while enhancing the well-being of older residents (16). The proportion of older people who are aged 60 and above is growing significantly (17). This is particularly the case for China where those aged over 60 comprise 264 million people, accounting for 18.7% of the total population (18). This number is expected to grow to 500 million by 2050. The country is also rapidly urbanising, with China's urban population growing from about 200 million in 1980 to about 800 million or 59% in 2018 (19). By 2030, the urban share of the population is expected to reach 70%, amounting to one billion urban residents (20) and of those urban populations, 1 in 4 will be older adults (21). The increasing ageing population is raising debates on how we can develop environments which best support older people to age well (22).

ICTs and smart cities are seen as having the ability to enhance active and healthy in older people by providing a creative and transformative approach (23). Combing smart cities and ICT technologies have the potential to provide a multi-dimensional and comprehensive solution to support older people in communities. The focus of such solutions is to support the creation and implementation of healthy, smart, and inclusive environments for older adults that enable them to actively participate in society while enjoying a healthy quality of life (23). Such solutions, which mainly include ICT-integrated smart homes (14), ambient assisted living (24) and home automation (25), are designed to facilitate active ageing and ageing-in-place through technological assistance. By creating new solutions and implementing best practise, the city with its aim of “go smarter” can optimise the potential of using the various capitals in cities and citizens, such as institutional, social and human capitals, and traditional (transport) and modern communication infrastructure (ICTs) (26), as well as integrating resources for supporting the participation of older people (23).

In response to the challenges of urbanisation and ageing, policy drivers, including WHO Age-Friendly Cities and Communities have explored how urban environments can deliver health and active ageing across key dimensions, including: (1) Outdoor spaces and buildings; (2) Transportation; (3) Housing; (4) Social participation; (5) Respect and social inclusion; (6) Civic participation and employment; (7) Communication and information; and (8) Community support and health services (27). Making cities and communities more age-friendly involves developing physical and social environments to meet the needs and requirements of older people while continuing to support older people to age-in-place (28). The WHO age-friendly framework have developed a number of associated guidelines and recommendations and the framework has been incorporated into region and country specific guidance e.g., WHO Europe and WHO Japan (29, 30). China has made similar progress, with Shanghai being a designated age-friendly city since 2006 (31) and recent announcements to launch 5,000 age-friendly communities by 2025 (32). In planning documents issued by the Shanghai Government (33), which aims to strengthen local age-friendly developments, it states that the local government will support the establishment of a IoT-related service platform for older people through providing online windows for government services. Additionally, the Shanghai government plans to build a number of standard age-friendly communities at a national-level by 2035 (34), in which it proposes to develop interconnected and sensory technologies in local communities. This will potentially enable local communities to interface with healthcare services and hospitals to assist in the development of age-friendly communities.

Despite this, there has been a lack of research explicitly linking smart city policy and age-friendly cities and communities. This is perhaps surprising, given the central role smart cities can play in developing interconnected monitoring technology (through the “Internet of Things”) that can provide opportunities for supporting the health and well-being of older people (35, 36).

### Building Linkages Between Social Inclusion, Ageing, and Age-Friendly Cities

Social inclusion is a key dimension of the age-friendly city agenda, underpinning the drive toward ageing in the “right” place which has highlighted the importance, not just of ageing at home and in the community, but having the resources and assets to enable full participation in old age (27). The concept of social inclusion focuses on how older people can achieve their full potential (37); whilst addressing equity and rights in respect of access to services, social cohesion and community participation, including feeling respected and valued (27, 38). Whilst the notion of social exclusion has been criticised for prioritising the deficits of old age (i.e., what older people lack), the concept of “inclusion” prioritises ageing as a positive process (i.e., what older people can be) (28, 39, 40). Here, the emphasis is on creating the conditions for enhancing individual “joining in” or “identifying” with the social world (41) rather than merely avoiding social isolation.

Significant research in environmental and social gerontology has focused on social inclusion in the context of ageing, older people, and their everyday environments (28, 39, 40, 42). An age-friendly community views social inclusion as one that ensures older people's meaningful roles in society and provides opportunities to access resources, maintain relationships, and meet basic needs (28, 43). Social inclusion can support the improvement of physical and social outcomes for older adults, ensuring enhanced quality of life in old age (39). Scharlach and Lehning (42) suggested that social inclusion for older people can be supported across five key areas: (1) continuity (i.e., absence of barriers to continued participation in long-standing activities and interests); (2) compensation (i.e., the ability to meet basic health and social needs in spite of age-related disabilities); (3) connection (i.e., opportunities to develop and maintain meaningful interpersonal relationships); (4) contribution (i.e., opportunities to participate in and have an impact upon one's social environment); and (5) challenges (i.e., development of stimulating new activities and interests). Scharlach and Lehning (28) go on to identify the key components of social inclusion for older people: reciprocal social exchange that promotes interdependence rather than inequity and disempowerment; social integration that supports social identity; role fulfilment and maintenance of self-construction and self-esteem; social recognition from community members and themselves; meaningful social interaction; and social agency rooted in mastery, self-efficacy, and perceived control of oneself and one's environment (28).

Levitas et al. (44) refers to social exclusion as being one of social deprivation, in terms of lack of integration in community, participation in community and civic life and exclusion from the benefits others are entitled to such as lifelong learning and education. Social inclusion encompasses multiple aspects of ageing, such as civic engagement, outdoor spaces, social participation and supporting an ageing workforce as forms of everyday inclusivity (45). In providing opportunities for social inclusion within the context of an age-friendly community, it also constitutes supporting meaningful roles for people in old age (46). Research has identified the importance of enabling social inclusion for older people within the context of the age-friendly city, building social participation and engagement, developing strong social capital and connections and developing a strong sense of place identity and attachment in old age (40, 47). Supporting social inclusion amongst older people is therefore recognised as a key priority and goal for the age-friendly city.

### Smart Technologies and Social Inclusion in the Context of Population Ageing

Smart technologies have been introduced in a number of ways at a home and community level to improve quality of life and independent living for older people, whilst offering opportunities for social inclusion (48, 49). At a city-level, technologies offer potential for the widespread diffusion of monitoring and sensor technologies to support transport delivery, mobility, and efficiencies in urban services *via* continuous and real-time monitoring (50, 51). In supporting an ageing population, Righi et al. (52) envisioned the potential for smart cities to deliver intergenerational urban communities through ICT interventions that are shaped around the interests and social practises of older people and which enable intergenerational connections to be formed. van Hoof et al. (35) identified how smart technologies can be deployed to support older people, e.g., health monitoring and emergency response systems, alongside assistance for activities of daily living in the context of the smart city. Others have highlighted the importance of scaling up what have been largely individually deployed interventions to date (e.g., body-worn sensor technologies) into an interconnected “city” scale approach thereby maximising what the smart city can offer (53). Likewise, advances in smart homes offer potential to support independent living, yet as van Hoof et al. (35) note, adoption is not widespread and lacks the connectivity across scales e.g., older people, carers, government, policymakers, which smart cities offer a potential framework for. As a result, smart city interventions to date have been limited in terms of realising their potential application for ageing and age-friendly cities.

In addition, there are a number of challenges in delivering technological products and services to older people (54, 55). Technological interventions have been relatively successful in responding to some of the functional needs of older adults, e.g., through medication reminders, but less so at delivering on broader challenges, e.g., those related to social inclusion including community engagement, social participation and equitability (56). Second, technological supports often fail to respond to the heterogeneity of the older person, both in terms of the ageing process and changing requirements across cultures in terms of what older people want from the technology (57). Third, the increasing Internet of Things (IoT) has raised new inclusion and equitability issues between who has access and who does not, and digital literacy which presents many from accessing online supports (58). The latter has been much discussed in the literature on smart cities and the digital divide (54, 55), pointing toward the “unevenly wired” and schisms between the “information rich” and “poor” (59). Reasons for the digital divide affecting older people vary but include: limited opportunities to accessing the internet (60); societal and individual attitudes impacting ICT use (49); physical health and learning disability barriers (61); socio-economic status and levels of deprivation (62) and differing interests in terms of what people want from the internet (63–65).

In summary, whilst there has been considerable policy rhetoric around the smart city, the role of older people, age-friendly environments and social inclusion in this agenda has received little discussion. Not only does this limit what can be said about ageing, smart cities and inclusion in empirical terms, but also prevents us from shaping smart “age-friendly” urban environments which deliver improved social inclusion and well-being for older people. In response, the aim of this paper is to explore the experiences of older adults living in a smart city in China and to understand how social inclusion amongst older people can be supported in relation to technology development and smart city intervention. This is underpinned by the following research questions: (1) how is ageing in place and social inclusion experienced by older people living in a case study city in China?; (2) how do older people perceive technology and smart cities within their everyday lives?; (3) how can smart city interventions support the social inclusion of older people through the development of age-friendly cities and communities?

## Methods

This study undertook a case study approach in Chongqing, China to capture the experiences of older people living in a smart city. The city of Chongqing is located in western China, and it is one of four municipalities that is administered directly by the central government [(66), pp. 43–44]. By the end of 2019, the total registered population in Chongqing was 31.24 million, of which 4.674 million people were aged 65 and over, accounting for 14.96% of the total population (67).

During China's period of rapid urbanisation, the Chinese state has strategically promoted various models of urban development e.g., eco cities and low-carbon cities (68). Over the last 10–15 years this has focused on smart city development. In 2013, the Chinese Ministry of Housing and Urban and Rural Development (MOHURD) announced that 193 Chinese cities had expressed a plan to “go smart” while approving nearly 300 cities to pilot the smart city concept (69) of which the case study city, Chongqing, was proposed as a key city. Subsequently, in 2015, the Chongqing municipal government launched a Master Plan for supporting Chongqing's Smart City development, 2015–2020, and in 2019, the “Chongqing New Smart City Construction Plan (2019–2022)” was developed (70). In the Smart City Plan of Chongqing, Yuzhong district was designated a smart city pilot district, aiming to provide impetus for other national and regional strategic smart cities (71). Taken into account its geographic location, population density, background in terms of economic and cultural development and ageing demographics, Yuzhong District was selected as the case study site for this research. There are 580,000 people living in Yuzhong District, with 120,000 people aged 60 and above, accounting for 19.76% of the total population within the district (72). Compared to other pilot smart city sites, Yuzhong District has the highest proportion of older people.

The study undertook initial pilot work in February and March 2019 to build an understanding of the case study context and to apply and refine the data collection instruments. The main fieldwork was undertaken across December 2019–January 2020. The participants in this research represent older adults aged 60 and above, across three communities in Yuzhong District of smart city Chongqing: Shiyoulu Community, Hualongqiao Community and Dahuanglu Community. Three communities were selected based on the learning from the site visits and place observations which were conducted as part of initial pilot work. In the observations, we undertook an audit of the community including for e.g., quality of outdoor spaces, services and amenities, and other aspects of the built environment. Combined with desk based work, we selected three communities representing diversity in terms of their spatial and physical characteristics, ageing populations, income (low, medium, and high), smart city development (implementation pathways), housing types, and other physical characteristics including built environment supports (see Table 1).

**Table 1:** Comparative information across three case study communities.

**Case study communities**	**Population**	**Population density**	**Population aged 65 years or over**	**Percentage of pop'n aged 65 years or over**	**Income**	**Public space and buildings**	**Smart city development**
Shiyoulu community	71,154	22,462/km^2^	5,773	8.1	Medium	- Mix of old and new residential and commercial buildings; - Improved physical environment (e.g., sufficient pedestrian crossings, anti-slip signs, visual signs); - Lack of green space and public seating; - Residential exposure to transportation noise - Restricted pavement and mobility space.	Smart community at the national level
Hualongqiao community	15,806	4,718/km^2^	1,390	8.8	High	- Urban redevelopment area with new and modern residential buildings, mixed type retaining many historical and cultural buildings; - Proximity to amenities and services, including cultural supports; - Clean and well-maintained public realm and green space;	Involved in the district smart city plan
Dahuanglu Community	81,658	36,110/km^2^	7,915	9.7	Low	- Housing stock is dense and of poor quality, with limited green space and utilities/amenities. - There is limited number of public spaces for social, cultural and commercial activities, but they require maintenance and refurbishment; - Walkability difficult and pavement/sidewalk barriers problematic.	Involved in district smart city plan

The focus of this research was to understand how social inclusion amongst older people can be supported through smart city development. The research design utilised multiple qualitative research methods, including semi-structured interviews (*n* = 69; 23 older people and 46 professionals), walk-along interviews (*n* = 21 older people), and focus groups (*n* = 20 older people spread across three focus groups), undertaken with older people and stakeholders of smart cities. A total of 64 older people engaged in the research across three selected communities (mean age 72.57, age range 60–90, with 25 male and 39 female) (see Table 2). Participants ranged in terms of gender, ages, socio-economic background (low, medium, and high levels of income), health status, education, living status, and household composition. A further 46 semi-structured interviews were conducted with smart city professionals involved in either ageing policy and practise or the delivery of the smart city agenda, including local government officers, technology companies, service providers, private companies, and care providers. In this paper, we present findings from the semi-structured interviews, walking interviews, and focus groups conducted with older people to better understand the experience of older people living in a smart city.

**Table 2:** Sample of participant older people's characteristics.

		**Chongqing, China**		
		**Dahuanglu community**	**Shiyoulu community (smart community)**	**Hualongqiao community**
Semi-structured interview		10	6	7
Focus group		6	8	6
Walking interviews		6	7	8
*N*:		22	21	21
Age				
	Mean	74.91	72.8	70.0
	Min.	60	60	62
	Max.	90	86	84
	Median	75	73	69
Gender				
	Female	13	16	10
	Male	9	5	11
Living arrangements				
	Living alone	2	2	6
	Living with others	20	19	8
Employment status				
	Retired	20	15	20
	Employed	0	6	1
	Volunteer job	0	0	1
	Unemployed	2	0	0
Years living in area (unit: years)				
	Min.	0.08	5	2
	Max.	50	60	62
	Mean	9.76	26.0	8
	Median	10	17	5
Income status (GBP)				
	Max.	£555.56	£1,111.11	£666.67
	Min.	£8.89	£111.11	£222.22
	Mean	£308.89	£356.08	£407.41
	Median	£333.33	£333.33	£333.33
Landlord registration				
	Landlord	15	16	12
	Not landlord	7	5	9
Education				
	No qualification	5	3	0
	Low	2	0	2
	Middle	5	15	13
	High	5	3	6

*1. Educational level is indexed on a four point scale (no qualification = non-educational; low = elementary education and lower vocational education; middle = secondary education and vocational education; High = college, university education, and scientific education)*.

*2. Referring to the exchange rate on 9 MAR. 2020, 1GBP equals to 9CNY*.

All the semi-structured interviews with local older residents were undertaken at the initial stage of data collection. All interviews were conducted in a place of choice for older people, with the older people's activity centre and public gardens being chosen by most participants as they represented safe and familiar environments for older people. After the semi-structured interviews with older people, participants were invited to undertake walking interviews and focus groups. Some older people chose to walk with the researcher after the interviews, while others undertook walking interviews after the workshops. A small number of older people did not wish to participate in either walking interviews or workshops due to health-related problems, mobility issues or because of their busy schedules. Focus groups were organised with local older people in each local community, and comprising 6–8 local older people in each group. The first focus group was held at the local community centre in Shiyoulu Community, the other two were held in local ageing care service centres. Local ageing care service centres are owned by private companies and ageing care institutes, in partnership with the local government. They are places which provide caring services and assistive technologies to support older people living in the local communities. Interviewees did not receive any reward for their participation in the study. All interviews were audio recorded.

All audio recordings were fully transcribed in both Chinese and English and prepared for full data analysis. The full interview transcription files were analysed in Nvivo12 through a thematic analysis approach using the six steps adapted from Braun and Clarke (73). The first phase involved reading and re-reading the transcriptions in order for the researcher to familiarise themselves with the data. The second phase involved coding the transcripts for initial themes, and organising the data into different groups and codes. The third phase involved searching for themes and considering how different codes may combine to form overarching themes. The fourth phase re-focused the themes and double-checked how they inter-relate to the coded extracts. Finally, each theme was defined and named. The resultant themes and quotations are used to support the findings of this research.

Prior to commencement of the study, a full ethics review was approved by Heriot-Watt University's School of Energy, Geoscience, Infrastructure and Society Research Ethics Committee. Before beginning data collection, informed consent was received from all participants. Participants were made aware of the research aim and objectives, what is expected from them in terms of data collection, and how the data would be recorded and re-produced. Issues of confidentiality and anonymity were discussed with older people. The data collected was safeguarded and stored in password-protected files.

### Findings

In order to understand how social inclusion amongst older people can be supported by the delivery of technological initiatives and smart city interventions, this research identified three key overarching themes through the interview data: (a) Challenges to Delivering Social Inclusion for Older People through Smart Cities; (b) Opportunities to Support Social Inclusion through Smart Cities; (c) Public Participation and (Dis)empowerment in Smart Cities; these were supported by five sub-themes (see Table 3).

**Table 3:** Themes and sub-themes from the thematic analysis.

**Overarching themes**	**Sub-themes**
Challenges to delivering social inclusion for older people through smart cities	Reinforcing social exclusion and inequality through smart cities
	Changing technologies, smart interventions and older people's requirements
	Insecurity arising from cybersecurity and privacy issues through using smart technology
Opportunities to support social inclusion through smart cities	Digital technologies to enhance social connectedness of older people
	Technology to support mental and physical health well-being of older people

*Public Participation and (Dis)empowerment in Smart Cities*.

### Challenges to Delivering Social Inclusion for Older People Through Smart Cities

#### Reinforcing Social Exclusion and Inequality Through Smart Cities

Research has identified some of the concerns about smart cities delivering inclusive social outcomes for urban citizens (74, 75). Our participants discussed a number of barriers and challenges to the deployment of smart technology in improving the lives of older people. Many expressed financial concerns around smart technology use. While older people recognised the importance of technology in supporting ageing in place and health and well-being in old age, worries over their financial security were seen as a significant barrier to adopting smart interventions. Here, older adults were concerned about technologies being available only for the “well off,” potentially widening existing societal inequalities in old age:

“I like smart technologies. They are great and important. I know it won't be a problem for those older people who have a high retirement income. But there are those who have a low retirement income, it will be a problem … it is a problem of financial income, certainly, the purchase of technology products are very expensive, the more intelligent products cost more. My family cannot afford it. You cannot say your family can afford; other families can, maybe he cannot.” (Male, 78, Hualongqiao Community)

In addition to income and financial insecurity, a number of interviewees were concerned that smart city interventions would also open up spatial inequalities, creating an urban (“well-connected”) and rural (“not well-connected”) divide. Given the significant socio-spatial inequalities between urban and rural communities in China (76), digital interventions have the potential to “exclude” vast numbers of older people from accessing supports:

“Yes, they are important [smart technology] and good. But I'm from a rural area, I don't have a retirement income, nor social pension. I'm living with my children and I eat whatever they buy for me. I have no income; I have no money. I cannot afford to buy these high-tech products.” (Female, 63, Dahuanglu Community)

Exacerbating these spatial and social issues, older people were also concerned about the extent to which smart cities would lead to the commodification of products and services targeted at and potentially exploiting older people. Research has identified concerns about the role of private companies in commodifying services and products which may target vulnerable groups in the application of smart cities (77). As a result, whilst many pointed toward the benefits of smart technology, older adults were concerned they would be “left out” of the smart city agenda as a result of their material circumstances:

“Yes, they are important [smart technologies]. It can measure blood pressure, locating where you are, and many other useful functions. But the quality has to be good too, to make sure of the accuracy of the result. But my biggest concern would be the price. I think they are very expensive. Most smartwatches cost 2000 Yuan (≈225 GBP), too expensive.” (Female, 79, Shiyoulu Community)

Older people also felt that levels of education would determine ability to use smart technologies with the “less educated” being excluded. Participants argued that level of education amongst older adults directly affects their ability and interest in technology use, challenges which have been well-documented in the literature (78). Due to the impact of past political influences, the Cultural Revolution in particular, a number of older people have traditionally been excluded from educational opportunities (79). Many felt they were not in a position to learn smart technologies and to develop the necessary knowledge around them. This had the potential to open up a cohort and class divide excluding the most vulnerable older adults from accessing smart interventions and excluding many from lifelong learning opportunities:

“You say that technologies, these are for people with higher educational background. Some older people can use it, but most of us cannot operate it. Especially people born in the 40/50s, we went to technical school at best. We do not enter university and receive higher education, we do not understand how to use technologies, we cannot use it [technology], cannot understand it [technology].” (Male, 75, Dahuanglu Community)

“That [technologies], of course, it requires a certain amount of knowledge, I have not received any education, I am an uneducated person, then we will certainly not use it [technology].” (Male, 65, Dahuanglu Community)

In addition, older people pointed toward a number of key challenges in implementing smart cities: (i) low technological take up amongst older adults preventing adoption of interventions; (ii) difficulties in perceiving how smart technology might bring about health and well-being benefits; and (iii) poor levels of participation and engagement amongst older people in the development of the smart city agenda:

“High-tech. we do not use these, and we do not understand what is that [smart technology]. Everyday of our lives, like today, is that we cook for ourselves, eating and watching TV ourselves. There is no high-tech. All day long, we do not participate in any social activities, and no one comes to inform us. (Female, 75, Hualongqiao Community)

“I think they are very important, all aspects are very helpful to older people. But I have not been heard about this, neither been exposed to the local development [of smart technologies]. I don't care about them, I don't use technology all the time.” (Male, 84, Hualongqiao Community)

In summary, our participants expressed a number of concerns about the deployment of smart cities and technologies, raising issues about their ability to bring about “inclusive” interventions for older people. In this sense, there remained considerable work to do in terms of reconciling smart cities with a socially sustainable agenda for older people in order to deliver opportunities for ageing in place.

#### Changing Technologies, Smart Interventions, and Older People's Requirements

In terms of older people's experience with accessing technology, older people argued that the design of technology did not often take into account the diverse requirements of older users. Many depended on others including family members to access online supports, raising concerns about the comprehensibility of technological supports, an issue which has been raised in the literature (80). A number of participants reported the need to be “navigated” through technology (a “digital pathway”) in order to access the services and supports they needed:

“Technology is actually very good for our older people, but we have no one to teach us and no one to guide us… My child helped me making an appointment for visiting GP, but I can't get a specialist number. I don't use online registration, I don't know how to do it. It's all because we don't have anyone to guide us.” (Female, 65, Dahuanglu Community)

Some older people reported that “*complicated”* and “*cumbersome”* technologies led to poor experiences when using technology, and often failed to support changing requirements in old age (81). Technologies and digital devices were seen as rapidly changing, which led to confusion and anxiety for many older people. Similar issues were encountered in terms of information and communication, with barriers to accessing technological supports in languages they could understand:

“All I think of this is that the smartwatch I've used before. It can measure blood pressure, heart rate and so on. I used that before, but it was so much complicated to use, so I threw it away. Even the language is also English. I am a Chinese speaker, and I don't know anything about English. How can I use that watch?” (Male, 68, Dahuanglu Community)

Other participants felt excluded from using technology as a result of physical disabilities and cognitive impairment, groups of older adults who are already amongst our most isolated and disconnected (82). For those experiencing declining cognitive function, accessing technology and digital devices was complex, with some lacking the social support and training available to provide assistance. As a result, the following older adult living with mild dementia, reveals the challenges of using technologies:

“My children come to visit me once a week. They usually give our lessons on using digital devices, like teach me how to use the phone. We want to learn how to use the smart phones like younger people do. But we cannot and it is very difficult to us to learn how to use. We, two old people, in the class we could understand some. However, every time after they leave, we forget quickly. We literally just forget how to use that device again. We always forget how to use it even though they already taught us.” (Female, 73, Shiyoulu Community)

In summary, whilst technologies often form a ubiquitous and pervasive aspect of our everyday environments, there remains barriers and challenges to their uptake, and which prevent them from being adopted as part of an integrated part of everyday life. These barriers are compounded by mistrust in using technologies, and insecurities which are further heightened by issues of privacy and data use, which we discuss in the following section.

#### Insecurity Arising From Cybersecurity and Privacy Issues Through Using Smart Technology

The impact of digital surveillance has been widely discussed in the research raising ethical and political issues related to the security of individual privacy and data management (83). This has become more acute in the context of smart cities, given the potential for continuous data monitoring across urban environments (84). At the same time, concerns around data surveillance have been heightened in China as a result of moves toward using smart interventions to monitor and potentially control behaviours (85). In the interviews, some older people raised concerns about how technologies and digital settings can address the issue of data exposure and protect personal privacy. The privacy of users and confidentiality were determined as the most important aspect impacting older people making decisions on whether to adopt smart technologies and monitoring in the home:

“Technology is important, but what if someone is monitoring me? I don't like being monitored and I won't agree to disclose my privacy.” (Female, 75, Dahuanglu Community)

Whilst in-home surveillance theoretically supported health care, security, and independence whilst living at home, some older people felt uncomfortable and insecure in relation to the monitoring of in-home activities. Many who had used technologies previously, felt their activities and movement were being monitored and restricted. This raised serious concerns within the context of smart cities, and the integration of real-time monitoring on a wider scale:

“I used security cameras before, I installed security cameras at home. It was originally used to monitor the kids, but instead put me under surveillance. I said to help me monitor now. Feel also embarrassed. I just don't like it. Why does it also monitor me? I don't like that … Imagine if this was on a city level. I don't want to disclose my private information to anyone.” (Female, 76, Dahuanglu Community)

Feeling insecure is also reflected in perceived mistrust in terms of who is controlling, accessing, and using the information of online technologies (86). Due to a lack of digital literacy, older people reported that it was difficult for them to manage online for fear of being exploited and anxieties of being “watched” and “controlled,” key concerns around surveillance and smart cities that have been identified in other research (87, 88). As a result, older people tended not to use the internet or digital access, calling for greater control over its deployment and use:

“Yes, the market needs to be better regulated and require security regulation supported by the government. Every time I use my phone and try to access website, I literally do not know which one I can click, which one I cannot. For example, many times I hear on the news that fraudulent companies specialise in targeting and scamming older people…. Those information online, what is trustworthy, and what is not reliable, I am an old woman, I don't know. Sometimes, I just click the pages in a randomness, then it shows up, then we are cheated and caught up in the scam.” (Female, 67, Dahuanglu Community)

In addition to the challenges of privacy and confidentiality in adopting technology, using technology and ICT at the scale of the city was new for many older people, resulting in a perceived lack of confidence in using smart technologies. Some older people expressed feeling anxious about using smart devices (“fear of getting it wrong”), heightening feelings of insecurity:

“For example, they say that we can give advice to the government online (e-governance), but I'm so afraid to click on those digital things. I actually envy those people who can use that (digital technology and application). But I'm not good in using those things and I can't use it. I have to learn, but I'm afraid I'll get it wrong and use it wrong. I have a headache when I think about using technologies.” (Female, 75, Shiyoulu Community)

Moreover, compared to the younger generation, older people had less experience and exposure to AI and other information technology, whether it is self-driving cars or mobile devices. As a result, older people felt that they are more likely to encounter barriers and psychological challenges to accessing digital systems. Others were concerned that technologies which are ill-thought through would not afford older people the safety and security that they needed in terms of trusting the technology:

“I've heard of self-driving cars, but I don't know how to operate them. And I think this car [self-driving] must not work in Chongqing, at least it needs another 10 years. The roads in Chongqing are very winding, climbing up and down, turning corners. Self-driving cars will take some time. And this technology is immature ah, there will be certain safety concerns. I'm afraid to use it now; after all, the technology is not mature.” (Male, 69, Hualongqiao Community)

Taken together, the themes above reveal many ethical challenges and barriers to delivering social inclusion for older people through smart city interventions. These issues point toward the need to reconsider and perhaps reconfigure notions of security, privacy, safety, and ethics in the context of smart city interventions in order to ensure older people feel safe and secure.

### Opportunities to Support Social Inclusion Through Smart Cities

#### Digital Technologies to Enhance Social Connectedness of Older People

Research has identified the potential social benefits and impacts of smart city interventions in terms of connectivity and mobility (89), but there has been no research exploring issues of social inclusion for older people. In identifying opportunities to improve social inclusion through smart city interventions, older people highlighted the importance of social participation and engagement as a key priority in terms of social well-being, including familial contact and social relations:

“Technologies, such as smart phone, are important for me in my everyday life. It makes my life convenient. I love to play Mahjong with my friends. Like today, I want to play Mahjong and meet my friends. I then called my friends directly. Ask them if they can make an appointment this afternoon and just come and play Mahjong together…. Also, I need to connect with my family…. You need to hang out, travel, contact friends and families. Keeping contact with friends and families are important to me in every day.” (Female, 65, Shiyoulu Community)

For many older people, smart cities have an important role to play in building connections and supporting older people to maintain meaningful interpersonal relationships. For example, in understanding what the main role of using mobile phones is for older people, participants emphasised that social companionship was important, through informal chatting, sharing images and news about day-to-day life events on social media platforms i.e., WeChat—a Chinese social media app.

In addition to the relational aspects of friendships and family, there was a specific role for technology in supporting everyday informal care in old age. This was related to activities of daily living e.g., sleeping, eating, and monitoring health and well-being alongside social support networks, suggesting a role for integrated smart technologies in linking informal care:

“The other thing is that my daughter has given me a new smartphone and everyday I use it to talk to my friends. My children will contact me on the phone. They will ask each other, how are you, what are you eating, did you sleep well last night? It's time to put on some clothes today. And which classmates ah, like our age, classmates are still around, so classmates in the phone shouted to catch up, then we go and meet up.” (Female, 87, Dahuanglu Community)

In addition to physical and social supports, staying connected to local services was deemed essential for older people and a key component of the age-friendly city, to ensure access to information and services. For older people, staying connected supported older people's sense of social engagement and feeling of security and safety:

“The ones I mentioned earlier, like travelling, contacting relatives, safety are all sprinkled in. I have a mobile phone, for example, so if something happens to me, I can call the hospital, or I can tell my relatives, or my friends, and it brings lots of convenience to me. Whether it's physically or mentally, it certainly gives me a fair amount of help and support.” (Male, 66, Hualongqiao Community)

Many participants identified the opportunities that technology can bring, reporting that using computers and mobile phones, particularly devices with interactive and communication-enabled applications brought opportunities to feel more included. Online networks can foster greater social interaction, particularly for those that are geographically disconnected (90). A number of older people in our study reported a sense of “being part of the outside world” as a result of technology, increasing happiness and well-being, and supporting ageing in place:

Interviewer: “Do you think that smart technology contributes to ageing?”

Participant: “It's very important. If you're connected to the internet now, you're basically connected to the outside world, and if you're connected, you don't feel lonely, because if you're at home alone, like you used to be, you'll get sick. If you're online now, you have more friends, you talk more and you're happier.” (Male, 60, Dahuanglu Community)

Participants also commented on the potential for using the internet and digital technology to access care services, medical advice and formal care in old age. Some older people felt that the internet and technology actively allowed older people to develop and maintain a close relationship with formal carers and healthcare practitioners. Thus, the smart city offers potential to deliver more meaningful interventions for older people if they are closely integrated with formal care (and in person, face to face) supports:

“In my case, that is to establish contact with community social workers and health care professionals to ensure good health. I have no one to take care of me and no children. If you get sick, you don't have anyone to take care of you, just like in some places, you die at home without even knowing. But here has volunteer services. The volunteers will come to visit once or twice a month. They will come to visit us once or twice a month and give you a talk, usually for an afternoon. They ask us what other needs we have, and they talk to us… you can call them if they are not feeling well. There is also a community-based family doctor who has come to our house a few times to see if we have any health problems, to see whether our blood pressure and blood sugar is high or not.” (Male, 90, Dahuanglu Community)

In building a sense of connectedness, older people reflected that beyond access to ICT and digital information, smart devices and websites have expanded sources of access to knowledge and information. Many reported having widened their interests in later life, developing their intellectual curiosity in terms of local and global affairs, and had a real desire to improve their personal skills, competency, and literacy in old age:

“There is a role for smart technologies, that is watching TV, for example. By accessing information online and TV can increase the breadth of our social news, information and knowledge” (Male, 69, Hualongqiao Community)

“This is very important, my generation is fine, we basically know how to use it, nowadays mobile phones are very important, we are contacting each other online. Reading news online and learning information online” (Male, 60, Dahuanglu Community)

Through using technologies in everyday life, older people realised their benefits in terms of establishing and sustaining relationship with caregivers, friends, and relatives. This provides much needed security in old age in terms of ageing in place. By accessing digital information and smart technologies, older adults could feel a proximity to people and services. Therefore, a key challenge for smart cities is how to establish online networks that enable older people to promote reciprocal social exchange and foster interdependence.

#### Technology to Support Mental and Physical Health Well-Being of Older People

For our participants, feeling secure also included having access to resources and knowledge to make their own decisions about their health and well-being. Older people reflected on the value of digital technologies in providing mechanisms to enhance opportunities for older adults with mobility limitations to use digital devices to maintain quality of life. Self-management of health and well-being through technology is seen as an important in determining independence and autonomy in old age (91) and in using smart products, i.e., smartwatch and smart beds, which have features to measure blood pressure and heart rate, allowing for everyday monitoring (92). Amongst our participants, older people felt that taking effective actions to respond positively to their health conditions was empowering and a sense of security came through having knowledge of that information and being able to respond to it. This was an important aspect of social inclusion for older people:

“You know, like mobile phones, those phones can measure blood pressure and all that. My husband has high blood pressure, so I just take the phone to test his body and use these devices to estimate if he has high blood pressure or not, and he knows what his blood pressure is. If his blood pressure is high, he then would take some medicine quickly.” (Female, 81, Dahuanglu Community)

“The economy is growing, technology has developed, and there are many benefits of using technologies. I see that the smart mattress can cheque blood pressure and physical fitness, which is very good, that brings lots of help for older people. Through these body tests, we can detect physical and health problems early and seek early medication and treatment from doctors.” (Male, 84, Hualongqiao Community)

Many older people we interviewed encountered social exclusion often as a result of their restricted mobility. Declining physical health gradually led older people to spend more time at home, resulting in feelings of isolation and exclusion. For many this was related to the absence of opportunities for transportation and mobility. Participants stressed the value of smart technologies in supporting the age-friendly agenda, for example, through supporting mobility and maintaining the home. In supporting everyday tasks and overcoming physical barriers, older people reported that smart technologies could free up more of their time and opportunities for social participation and civic engagement. Despite this, many were still to see any real benefits or smart city applications in their everyday lives:

“The important thing is that you can buy a self-driving car, for example, if you want to go somewhere, you tell the car (self-driving car) and it comes to pick you up. Also, when the car arrives at our destination, the car will automatically notify us to get off.” (Male, 75, Dahuanglu Community)

“If features of smart technologies can be achieved and applied to older people, of course, then these can help… such as robots can sweep the floor, do housework for us, those features are very good. But now we have not seen those things happened to us, our community does not seem to have seen those robots sweeping the floor.” (Male, 70, Shiyoulu Community)

While older people reported feeling insecure and uncomfortable due to in-home surveillance, older people did report a flip side to this, in terms of enhancing security and safety. Smart technologies had a potentially positive role to place in enhancing feelings of security e.g., through real-time detection, hazard warnings, emergency response, as well as everyday reminders. The fact that older people could “monitor and see” the technology was important:

“If it's a smart home, if it's a burglar that comes into the house, the system will warn you and the police. Sometimes it also reminds you, for example if you go out and forget your keys, the smart device will remind you. It's just nice and convenient. If I put a camera in the house, at night when I'm sleeping, I turn this on at night. If a burglar comes in or something dangerous happens, I can know about it, and I can monitor it, I can see it.” (Female, 87, Dahuanglu Community)

In addition to physical supports, older people also reported on the importance of mental health and well-being in old age. For many, smart technologies did not necessarily need to be integrated into homes and buildings to bring about benefits. The use of portable products such as mobile phones, smartwatches, wearable devices and robots had the potential to deliver advantages. For many, smart technologies for older people afforded a feeling of “companionship” and comfort in old age. Here, it was important that technology was able to develop a two way relationship with the older person, offering a range of physical and social benefits:

“I have a robot, and there is a robot in my house. It's called “Meihao.” Every time you shout “Meihao, Meihao! I want to listen a storey.” and it will tell you a storey… But now it doesn't work because it needs internet support, it doesn't work without internet support. It's connecting to the internet, and when it's connected, it's ready to use. Call him to sing to you, and he will sing to you. When you are not feeling well, you can ask him what medicine you need to take. It will also tell you. He says, “You should go to the hospital and see a doctor.” Then I would go to the hospital.” (Male, 75, Dahuanglu Community)

For some, older people felt smart environments had the potential to support their everyday life by being able to diagnose and intervene in response to health conditions, which has been a key area in the development of technology for older people (93). Through connecting to medical services and GPs, smart devices facilitated more support, flexibility, and convenience in the lives of older adults:

“If you are sick, you have to tell him (e-health service). The doctor will answer you, which medicine you should take, which place you should go, where you are not well. I went to buy medicine this afternoon and there was a computer doctor at the Pharmacy. When you see the computer doctor you just have to tell him what is wrong with you and where you are not well? The computer doctor will then tell you what medicine to take.” (Male, 81, Dahuanglu Community)

In summary, smart technologies can provide opportunities for improving the social inclusion of older people, through supporting everyday tasks, enhancing a sense of security and facilitating social participation. Through improved connectivity, older people can receive physical support through using digital technology to maintain social connections, and ultimately contribute to the social inclusion of older people, improving access to supports and resources to age well.

#### Public Participation and (Dis)Empowerment in Smart Cities

Participants reflected on the issues of involvement and participation in the smart city agenda. In discussion on the progress of smart city development, older people reflected that there is neither the chance to participate in local development, nor has there been tangible improvements as a result of smart city initiatives. Whilst many had heard of smart cities, older people felt that smart city development had afforded little impact on their everyday lives:

Interviewer: “Do you know there is a smart community in Shiyoulu, Yuzhong District? Can you feel the change?”

Participant: “Yes. We know it. Our district has one as well. I heard that our universities and governments spent 30 million to build one. But I haven't felt any changes.” (Female, 75, Shiyoulu Community)

There were few opportunities for older people to input into the decision-making process within communities, reflecting a lack of formal engagement opportunities as part of the smart city agenda. The lack of citizen engagement in smart cities has been noted as a shortcoming in the literature (94). Many felt as if they were not listened to and lacked knowledge of where to go to in order for their voice to be heard:

Interviewer: “Can you make advice or suggestions?”

Participant: “No. Nobody listens. Nowhere to speak, report and appeal. We cannot find that place.” (Male, 67, Shiyoulu Community)

Due to the lack of consultation and engagement between older people and government, participants expressed a feeling of distrust toward the government. Many were sceptical as to the extent to which the policy around smart cities would be translated into actual practise:

“Now the old people mostly complain that the government do not follow their words. They say one thing but do another.” (Female, 75, Shiyoulu Community)

In having their voice heard, many felt that there was a stigma around ageing and older people, with their opinions and expertise being afforded lower priority than others in the smart city agenda. Participants reported a sense of helplessness, reflecting on their perceived lack of value to the local community, and a feeling of marginalisation:

Interviewer: “What do you think is the best way for older people to improve the current problems?”

Participant: “Nothing needed. An old person can do nothing. Others dislike older people.” (Female, 83, Hualongqiao Community)

“It is nonsense to participate in the local development, because nobody wants to hear our voice. Nobody really cares what we say whenever we give suggestions and comments on developments such as the smart city. We've been marginalised. Who cares about you? No one care about you.” (Male, 67, Shiyoulu Community)

Participants emphasised the value of participation in the community in terms of being informed and aware about what is happening. In the types of participation which could be better supported through the smart city agenda, older adults specified both online and offline. For those experiencing mobility challenges, then online participation provided an opportunity to participate, providing they had the technology and means to do so. For others, collective participation through in-person and face to face engagement was important. In all cases, engagement and participation in the smart city agenda were seen as integral to feeling a sense of purpose and citizenship in society:

“So participating in making smart city policies or getting involved with the society should be accessible from both online and offline.” (Female, 87, Dahuanglu Community)

“I think so. It can broaden our views. It would be convenient. And we can have a better involvement with society, which is also a kind of way to participate in society.” (Male, 84, Hualongqiao Community)

In addition to having the opportunity for older people to participate in the planning process, many reported on the need to ensure that the experiences of older people are incorporated into the smart city agenda. By involving multi-agency groups and engaging rights and advocacy organisations in the smart city agenda, then the rights and interests of older people can be increasingly protected. To others, the role of older people as community leaders was central to developing smart city interventions that reflected the requirements of older people. An enhanced role for older people's champions as advocates for change was important in delivering meaningful interventions:

“Then we need the community leaders to manage the community well. They can lead us and guide us. If they do not manage the community, then no matter what things we do is meaningless.” (Male, 67, Shiyoulu Community)

For others, participation in the smart city agenda was closely related to the quality of engagement with services. Although older people felt that accessing services and information online was important, smart technologies cannot replace manual and face-to-face service delivery. The importance of “local navigators” was crucial here, having offline services and guidelines available in the local community, advising people through the technologies and services to facilitate access for older people:

“Those digital services are good, I know. But the problem is that we don't know how to use it. For example, if you want to take a taxi outside, you have to book it online, because there are many internet cars now, we, older people don't know how to take that digital taxi. So we don't even take a taxi because we don't know how to use that app and there's no one to teach us, no one to guide us. For example, if you don't know how to use it, it would be helpful if the community or the platform could have someone around to teach you how to use the application and guide you when we are using these services. Without someone to teach us, we don't know how to use them, and we won't use it.” (Female, 76, Dahuanglu Community)

Perceptions of participation and engagement raise critical questions for the smart city and ageing agenda. A lack of opportunities to participate in smart city development could potentially exclude older adults from the decision-making process resulting in disengagement and disillusionment with smart cities. In our participant accounts, this was linked to feelings of disempowerment and disenfranchisement, as well as undermining their sense of citizenship in the smart city. Building trust and reciprocity among government, service providers and older people is an essential step toward developing inclusive smart age-friendly cities. Different forms of participation are needed to reflect the desire and ability of older people to participate in different ways. Likewise, the voice of older people needs to be shared and heard in a more meaningful way, prioritising their experiences of living in communities.

## Discussion

In this study, we sought to explore experiences of older people living in a smart city and discuss how social inclusion amongst older people can be supported in relation to technology development and smart city interventions. Through the five key themes presented in this paper, we have explored the role of smart city development in the lives of older people and its ability to support the ageing in place requirements of older people. Our findings revealed there is the potential for technology and smart city interventions to address some of challenges of an ageing society (23). For example, in maintaining and supporting strong familial connexions while strengthening the social participation of older adults. Likewise, smart cities can potentially provide opportunities to access health information online to enhance self-health management and well-being. Yet smart cities also bring about challenges that need to be overcome in order to support the inequities of ageing across urban environments. In some cases, the smart city agenda and digitization more broadly has the potential to reinforce urban inequalities, through inconsistencies in access to technology, thereby creating a digital divide and enhancing social exclusion (58). There was a deep misunderstanding and mistrust amongst older people regarding the use of the smart city and its aim e.g., surveillance. Furthermore, many feel excluded from the smart city agenda, excluded from urban place-making practises and the development and deployment of smart city technologies. In order to deliver smart, “inclusive” environment for older people, active participation and empowerment of older people should be considered as a priority in smart city development. In doing so, this discussion points toward some specific recommendations for ageing and smart city theory, policy, and practise moving forward, if it is to deliver socially equitable and age-friendly outcomes for older people. We bring together the findings from the research with the burgeoning literature on age-friendly cities and communities to identify potential ways forward.

### Theories of Ageing, Place, and Technology

Theories of environmental gerontology have explored older adults relationship with their environment, building on notions of person-environment fit to explore the extent to which everyday settings e.g., home and community, support changing contexts in old age (95). More recently, this has included an appreciation of the relational, interconnected, and interdependent ways in which older people form attachments with their immediate environment (47, 96). At the same time, critical gerontology has identified the disconnect between technological interventions and ageing-in-place, citing that technological supports often lack the ability to deliver on forms of social participation and community integration which are integral to ageing-in-place (97). Going forward, further transdisciplinary work is needed to bridge theories of gerontology with smart city discourse, to explore how we can better integrate notions of ageing-in-place in smart cities. If we are to deliver smart urban environments that support older people, then we need to ensure that such technology is able to build relational aspects of place in the lives of older adults, where smart city interventions enable social participation, community participation and civic engagement. Likewise, there is a need to learn from smart city theory to examine how we can address issues of surveillance, empowerment, and rights to the city in the context of age-friendly environments. For example, theories of smart citizenship (98) offer valuable theoretical frameworks for conceptualising issues of power in the context of the smart city and emphasise the need to challenge top-down models of smart cities. This is important if we are to support a rights and citizenship agenda, where older people are central to driving forward smart city interventions.

### Integrated Smart City-Age-Friendly Policy Discourse

Whilst social inclusion was considered important in supporting health and quality of life for older people, and many could see the potential for technological supports to enable ageing in place, there is no existing interconnected policy framework for ageing and smart city policies in China. Given the expansion of the smart city movement and the rapidly ageing society in China, closer integration of these agendas is important to realise the potential of smart urban environments in supporting positive outcomes in old age. Age-friendly interventions already offer a potential framework through which to connect the vision of ageing and social inclusion, of which smart cities and technology should be a cross-cutting strand. Important here, is realising and joining up the smart city agenda with each of the various dimensions identified in the WHO (27) age-friendly framework: (1) Outdoor spaces and buildings; (2) Transportation; (3) Housing; (4) Social participation; (5) Respect and social inclusion; (6) Civic participation and employment; (7) Communication and information; and (8) Community support and health services, so that there is a cross-cutting and holistic approach to bringing about change. This requires more integrated and joined-up solutions (41) that establish how smart cities can “speak to” and address some of the key challenges of an ageing population, e.g., housing, outdoor spaces, health and well-being, participation and engagement. Failing to deliver integrated solutions, will likely continue to see smart cities and ageing as separate strands of urban and social policy, creating silos and disconnected practise, which will fail to deliver the wraparound digital supports that older people need.

### Ageing, Social Inclusion, and Connected Communities

Participants identified what they wanted from smart city technologies in terms of enhancing their social participation and inclusion, highlighting the importance of residing in connected communities when ageing in place. This involved three specific domains and criteria for the smart city in terms of enabling physical, social, and community connectivity. These were integral to personal development, maintaining interdependent relationships in old age, and supporting well-being and quality of life. Physical connectivity related to the direct engagement with services and service providers, enabling people to access services as and when they need them. Social connectivity related to how smart technology can facilitate social relations between family and friends, as well as informal social support networks to support everyday health and well-being. Community connectivity relates to the wider engagement of older people with the community, in terms of opportunities for participation and meeting others, i.e., involvement in community life. These three domains represent important areas of priority for smart cities, in terms of delivering on socially inclusive age-friendly communities for older people which support relational connections in old age and create truly interconnected and interdependent communities.

### Inclusive Smart Cities and Widening Participation

Whilst ICT-based technology had the potential to bring benefits to older people's everyday life, there were a number of challenges to using digital technologies. These concerned: (i) financial constraints, (ii) digital literacy, and (iii) health and cognitive issues in old age which prevented older groups from being able to access digital supports. These exclusionary barriers have the potential to widen social inequalities and undermine the social inclusion agenda, compounding isolation and exclusion for the more “hard to reach” older adults. These issues have also been supported by Fang et al. (58) and Lee et al. (99) in the literature. As technology is becoming a more pervasive part of the urban environment, then services and supports are becoming increasingly digitally mainstreamed. Our participants highlighted serious concerns and anxieties regarding access to digital services. For those that are digitally connected, then realising those opportunities for active and healthy ageing will become more feasible in the context of a smart city. Educating older people to improve their digital literacy can potentially improve mental and social well-being (99). In contrast, for those who are digitally excluded, there is the danger that the inability to access supports will open up new health and well-being inequalities. Smart cities need to ensure that technologies are accessible for all, are inclusive (regardless of financial means) and that digital literacy becomes a central component of delivering interventions. Moreover, technological change can be overwhelming for older people, in the same way that housing transitions and other sudden societal changes can lead to negative outcomes in old age. Managing digital change and transitioning it effectively in the lives of older people is important if we are to realise the benefits of smart technologies.

### Rights, Ageing, and the Smart City

In delivery of smart cities, two critical issues were raised in the context of rights and the digital city. In terms of governance of smart city initiatives, many were concerned about the ubiquitous use of technologies in monitoring the public and private life of older people. Participants expressed concerns over what data was being collected, how that data was being used and who controls and owns that data (83). This is perhaps more pertinent given the case study context and the increasing surveillance in China which has been well-documented (85). As smart cities have the potential to establish varied and complex digital connections across the city, there are concerns over its intrusion into private life. Here, the smart city agenda needs to ensure that autonomy, choice, and control are supported (81), involving older people in decision-making about how data is being managed and utilised. Too often, people are presented with a “privacy trade off” in being promised more efficient services, yet in supporting the health and well-being of older people, there should be clear transparency over monitoring technologies and the data that is being monitored. A related point here is the danger of creeping privatisation and commodification of services as part of the smart city (8), which many of our participants were concerned would lead to the exploitation of older people. The smart city agenda needs to ensure that the notion of rights is reconfigured in the context of smart technology, ensuring that the rights of older people to age in place are forefronted (e.g., right to move around urban spaces, right to safe and secure housing, right to employment opportunities), whilst balancing a set of rights in the context of technology and data monitoring e.g., the right to privacy, security, and safety. In configuring these rights, there needs to be a central role for older people's advocacy groups, NGOs and community stakeholders, alongside the public and private sector, in multi-agency partnerships built upon good governance and ethics, which ensures a rights based approach to the provision of supports for older people in the context of the smart city.

### Co-producing Smart Age-Friendly Cities

Our findings pointed to a lack of information and knowledge amongst older people about the notion of the smart city and technologies associated with it. For many, smart cities were seen as empty policy rhetoric. This suggests that significant work needs to be undertaken around the smart city agenda to engage older people. Much smart city research has criticised its implementation in terms of top-down approaches to technological implementation. This has had the impact of alienating older people who do not talk “the policy language” from engagement in constructing what smart cities are and was deeply disempowering for our participants. As a result, thus far smart city policy has little relevance to older people and the practise responses have failed to address their everyday lives. Smart cities need to ensure that older people are (i) informed and realise the benefits of smart city interventions and (ii) are actively involved as partners in the design and deployment of the smart city agenda. Producing digital products that are responsive to the needs of the users will not only increase the acceptance of older people in a technology-led society, but will also bring positive outcomes for older people as technology is shaped around the lives of older people. There is much we can learn from the age-friendly city and community movement here regarding wider citizenship in the context of the smart city. For example, age-friendly champions and older people's forums have been fundamental in delivering on the citizenship aspect of the age-friendly agenda, where older people are at the centre of policy-making decisions. Similar empowering practises are needed to ensure that the voice of older people is used in a positive way to drive a smart, age-friendly agenda.

## Conclusion

This research adopted a qualitative case study approach to investigate the experience of older people living in a smart city in China, and to discuss how technological initiatives and smart city interventions can support the social inclusion of older people. Our findings revealed the opportunities and challenges of supporting social inclusion amongst older people living in a smart city. In the research, we specifically focused on the lived experience of older people across three communities in a smart city in Chongqing, China. In terms of opportunities, interconnected smart technologies at a city scale, can deliver potential positive health and well-being outcomes, and our participants were optimistic about the role of smart cities in supporting age-friendly urban environments through deeper physical, social, and community connectivity. Yet, there exist a number of challenges to delivering improved social outcomes for older people, including how smart technologies can deliver improved autonomy, choice and control, as well as ensuring that smart interventions are equitably delivered including the need to: enhance social participation and social supports for all; supporting role fulfilment and changing identities in old age; and encouraging interdependence among older adults. Lastly, closer reconciling of the age-friendly agenda with smart city policy and practise is needed to ensure changes at the city level deliver promised well-being and quality of life benefits in old age. This is critical if smart cities are to respond to ageing societies and realise their potential role in delivering positive social outcomes for older people.

### Limitations and Implications for Future Research

We conclude by highlighting some strengths and limitations of the work. This works draws upon experiential case study research in specific communities in Chongqing, providing insight into perceptions of smart cities and ageing-in-place amongst older people. Whilst this has addressed an important gap, more research is needed to explore perspectives across different smart city case study locations in China and globally (including the Global South) and their impacts on the ageing population. This would enable us to build up a more comprehensive understandings of smart city impacts across different city planning and governance frameworks. In terms of sampling, we do draw upon a diverse range of experiences by age, gender, and place, but it was outside the scope of this study to undertake an analysis of that data by specific age cohorts, although we recognise this is needed in order to capture the diverse experiences of different age groups in relation to age-friendly cities and attitudes to technology and smart cities which are known to differ by age (young-old; old; and the old-old). Likewise, the sample did not fully capture older people across various categories e.g., cognitive decline, although there is reason for us to believe that this impacts on technology use and the types of age-friendly interventions required at a community and city level to support ageing in place. Lastly, in collecting the data as part of this study, it was complex for older people to visualise the notion of the smart city. Prompts were often needed in focus groups, such as examples of smart city initiatives, to elicit discussion. This speaks to the need for closer involvement of older people in the smart city agenda, through city initiatives which directly engage older people to ensure they are more informed and aware of what constitutes the smart city, but which nevertheless also speaks to a weakness in the data collected.

## Data Availability Statement

The raw data supporting the conclusions of this article will be made available by the authors, without undue reservation.

## Ethics Statement

The studies involving human participants were reviewed and approved by Heriot-Watt University's School of Energy, Geoscience, Infrastructure and Society Research Ethics Committee. The patients/participants provided their written informed consent to participate in this study. Written informed consent was obtained from the individual(s) for the publication of any potentially identifiable images or data included in this article.

## Author Contributions

ML and RW: conceptualisation and methodology. ML: investigation, data collection and analysis, writing—original draught preparation, and visualisation. RW: review, editing, and supervision. Both authors have made significant, direct and intellectual contributions to the work, and approved the submitted version for publication.

## Funding

This study was supported by The School of Energy, Geoscience, Infrastructure and Society (EGIS) at Heriot-Watt University.

## Conflict of Interest

The authors declare that the research was conducted in the absence of any commercial or financial relationships that could be construed as a potential conflict of interest.

## Publisher's Note

All claims expressed in this article are solely those of the authors and do not necessarily represent those of their affiliated organizations, or those of the publisher, the editors and the reviewers. Any product that may be evaluated in this article, or claim that may be made by its manufacturer, is not guaranteed or endorsed by the publisher.

## References

[1] Castells M. Communication, power and counter-power in the network society. Int J Commun. (2007) 1:29.

[2] Tonkiss F. Cities by Design: The Social Life of Urban Form (2014). John Wiley & Sons. Available online at: https://books.google.com/books?hl=en&lr=&id=fDGpAgAAQBAJ&oi=fnd&pg=PT6&dq=Cities+by+design:+the+social+life+of+urban+form.+John+Wiley+%26+Sons&ots=Kxd5CHTUl9&sig=DtxLBkK6cDuIkmeV33mm32I67UQ (accessed September 10, 2021).

[3] Nam T Pardo TA. Conceptualizing smart city with dimensions of technology, people, and institutions. In: Proceedings of the 12th Annual International Digital Government Research Conference: Digital Government Innovation in Challenging Times (2011). p. 282–91.

[4] Albino V Berardi U Dangelico RM. Smart cities : definitions, dimensions, performance, and initiatives. J Urban Technol. (2015) 22:3–21. 10.1080/10630732.2014.942092

[5] Huovila A Bosch P Airaksinen M. Comparative analysis of standardized indicators for smart sustainable cities: what indicators and standards to use and when? Cities. (2019) 89:141–53. 10.1016/j.cities.2019.01.029

[6] BSI. Smart Cities Framework – Guide to Establishing Strategies for Smart Cities and Communities (2014). Available online at: https://www.bsigroup.com/en-GB/smart-cities/Smart-Cities-Standards-and-Publication/PAS-181-smart-cities-framework/ (accessed August 29, 2021).

[7] Shelton T Zook M Wiig A. The ‘actually existing smart city’. Cambridge J Reg Econ Soc. (2015) 8:13–25. 10.1093/cjres/rsu02615366237

[8] Marvin S Luque-Ayala A McFarlane C. Smart Urbanism: Utopian Vision or False Dawn? London: Routledge (2015). 10.4324/9781315730554

[9] Green B. (City Planner). The Smart Enough City : Putting Technology in Its Place to Reclaim Our Urban Future. MIT Press (2019). Available online at: http://mendeley.csuc.cat/fitxers/e78753da0384026d087b39ba2c9c6218 (accessed September 9, 2021).

[10] Li Y Lin Y Geertman S. The development of smart cities in China. In: CUPUM 2015 - 14th International Conference on Computers in *Urban Planning and Urban Management*. Cambridge, MA (2015).

[11] Brdulak A. The concept of a smart city in the context of an ageing population. Zesz Nauk Uniw Gdańskiego Ekon Transp i Logistyka. (2017) 68:65–75. 10.5604/01.3001.0010.5323

[12] Vanolo A. Is there anybody out there? The place and role of citizens in tomorrow's smart cities. Futures. (2016) 82:26–36. 10.1016/j.futures.2016.05.010

[13] Cardullo P Kitchin R. Being a ‘citizen’ in the smart city: up and down the scaffold of smart citizen participation in Dublin, Ireland. GeoJournal. (2019) 84:1–13. 10.1007/s10708-018-9845-8

[14] Klimczuk A Tomczyk L. Smart, age-friendly cities and communities: the emergence of socio-technological solutions in the Central and Eastern Europe. In: Active and Assisted Living. (2016). p. 335–59.

[15] Phillipson C. Developing age-friendly communities: New approaches to growing old in urban environments. In: Handbook of Sociology of Aging. New York, NY: Springer (2011). p. 279–3.

[16] Keng Hua Chong Mihye Cho. Creative Ageing Cities: Place Design With Older People in Asian Cities. Routledge (2018). Available online at: https://books.google.co.uk/books?hl=en&lr=&id=uilKDwAAQBAJ&oi=fnd&pg=PT15&dq=info:4jCOMJ6itaYJ:scholar.google.com&ots=HkHZoo1jIw&sig=PAnqmBbT5W2lVy5mtq-U0_rM0Z0&redir_esc=y#v=onepage&q&f=false (accessed October 25, 2021).

[17] WHO. Ageing and Health. World Health Organization (2018). Available online at: https://www.who.int/news-room/fact-sheets/detail/ageing-and-health (accessed September 9, 2021).

[18] National Bureau of Statistics. The 7th National Population Census (2021). Available online at: http://www.stats.gov.cn/tjsj/zxfb/202105/t20210510_1817176.html (accessed August 24, 2021).

[19] Hamnett C. Is Chinese urbanisation unique? Urban Stud. (2020) 57:690–700. 10.1177/0042098019890810

[20] UN DESA. The World Population Prospects: 2015 Revision. United Nations Department of Economic Social Affairs (2015). Available online at: https://www.un.org/en/development/desa/publications/world-population-prospects-2015-revision.html (accessed August 30, 2021).

[21] UN. World Population Ageing (2015). Available online at: https://www.un.org/en/development/desa/population/publications/pdf/ageing/WPA2015_Report.pdf (accessed September 9, 2021).

[22] Phillipson C. Urbanisation and ageing: towards a new environmental gerontology. Ageing Soc. (2004) 24:963–72. 10.1017/S0144686X0400240530886898

[23] van Staalduinen W Dantas C van Hoof J Klimczuk A. Building inclusive environments for all ages with citizens. In: In Sheldon 3rd Online Conference Meeting: Solutions for Ageing Well at Home, in the Community and at Work (2021). p. 11. Available online at: https://papers.ssrn.com/abstract=3942789 (accessed October 24, 2021).

[24] Bronzini F Barbini N Marinelli G Pantaloni M Giordano E. Active ageing and public space: the creative city 3.0. In: Longhi S Siciliano P Germani M Monteriù A editors. Ambient Assisted Living. Cham: Springer (2014). p. 361–71.

[25] Hui TKL Sherratt RS Sánchez DD. Major requirements for building Smart Homes in Smart Cities based on Internet of Things technologies. Futur Gener Comput Syst. (2017) 76:358–69. 10.1016/j.future.2016.10.026

[26] Bifulco F Tregua M Amitrano CC D'Auria A. ICT and sustainability in smart cities management. Int J Public Sect Manag. (2016) 29:132–47. 10.1108/IJPSM-07-2015-0132

[27] WHO. Global Age-friendly Cities - A Guide (2007). Available online at: www.who.int/ageing/enFax:+41 (accessed December 19, 2018).

[28] Scharlach AE Lehning AJ. Ageing-friendly communities and social inclusion in the United States of America. Ageing Soc. (2013) 33:110–36. 10.1017/S0144686X1200057830886898

[29] EU. Towards an Age-Friendly European Union By 2020 (2012). Available online at: https://www.age-platform.eu/sites/default/files/Towards_an_Age_Friendly_EU_FINAL.pdf (accessed October 27, 2021).

[30] Aung MN Koyanagi Y Ueno S Tiraphat S Yuasa MAC. A contemporary insight into an age-friendly environment contributing to the social network, active ageing and quality of life of community resident seniors in Japan. J Aging Environ. (2021) 35:145–60. 10.1080/26892618.2020.1813232

[31] Fang L Wang J Zhang W. A case example of research to plan age-friendly action in Shanghai, China. Innov Aging. (2017) 1:39–40. 10.1093/geroni/igx004.156

[32] CGTN. China to Create 5,000 Age-Friendly Communities From 2021 to 2025 - CGTN. CGTN (2020). Available online at: https://news.cgtn.com/news/2020-12-06/China-to-create-5-000-age-friendly-communities-from-2021-to-2025-W079vI5Vja/index.html (accessed October 27, 2021).

[33] Shanghai Government. The Issuance of the “14th Five-Year Plan” for the Development of the Elderly in Shanghai. Shanghai Munic People's Government (2021). Available online at: https://www.shanghai.gov.cn/nw12344/20210616/10f25167e2fd4e4ca9f1a09dd80dee0e.html (accessed October 27, 2021).

[34] WSJKW. Notice on the Creation of Age-Friendly Communities in the City__Shanghai Health Care Commission. Shanghai Municipal Health Commission (2021). Available online at: http://wsjkw.sh.gov.cn/gjhztgahz/20210315/6b801b789ae046dc8485ec7256d4b4c9.html (accessed October 27, 2021).

[35] van Hoof J Kazak JK Perek-Białas JM Peek STM. The challenges of urban ageing: making cities age-friendly in Europe. Int J Environ Res Public Health. (2018) 15. 10.3390/ijerph15112473 30714577 PMC6266083

[36] Li M Woolrych R. Exploring the disconnection between smart city policy and practice and social inclusion amongst older people. In: AMPS Proceedings Series 21. Rapid Cities – Responsive Architectures. American University in Dubai. UCL Press (2020). p. 311–9.

[37] Office of the Deputy Prime Minister L. A Sure Start to Later Life: Ending Inequalities for Older People (2006). Available online at: www.socialexclusion.gov.uk (accessed December 24, 2020).

[38] Bure C. Digital inclusion without social inclusion: the consumption of information and communication technologies (ICTs) within homeless subculture in Scotland. J Community Informatics. (2005) 1:116–33. 10.15353/joci.v2i2.2078

[39] Buffel T Phillipson C Scharf T. Experiences of neighbourhood exclusion and inclusion among older people living in deprived inner-city areas in Belgium and England. Ageing Soc. (2013) 33:89–109. 10.1017/S0144686X1200054230886898

[40] Woolrych R Sixsmith J Fisher J Makita M Lawthom R Murray M. Constructing and negotiating social participation in old age: experiences of older adults living in urban environments in the United Kingdom. Ageing Soc. (2021) 41:1398–420. 10.1017/S0144686X1900156930886898

[41] Haddon LG. Social exclusion and information and communication technologies: lessons from studies of single parents and the young elderly. New Media Soc. (2000) 2:387–406. 10.1177/1461444800002004001

[42] Scharlach AE Lehning AJ. Creating Aging-Friendly Communities. New York, NY: Oxford University Press (2016).

[43] Spina J Menec VH. What community characteristics help or hinder rural communities in becoming age-friendly? Perspectives from a Canadian prairie province. J Appl Gerontol. (2015) 34:444–64. 10.1177/0733464813496164 24652896

[44] Levitas R Pantazis C Fahmy E Gordon D Lloyd E Patsios D. The multi-dimensional analysis of social exclusion. Lloydia Cincinnati. (2007) 1–246.

[45] Warburton J Ng SH Shardlow SM. Social inclusion in an ageing world: introduction to the special issue. Ageing Soc. (2013) 33:1–15. 10.1017/S0144686X1200098030886898

[46] Barrett P Hale B Gauld R. Social inclusion through ageing-in-place with care? Ageing Soc. (2012) 32:361–78. 10.1017/S0144686X1100034130886898

[47] Woolrych R Duvurru J Portella A Sixsmith J Menezes D Fisher J. Ageing in urban neighbourhoods: exploring place insideness amongst older adults in India, Brazil the United Kingdom. Psychol Dev Soc J. (2020) 32:201–23. 10.1177/0971333620937106

[48] McPherson BD Wister A. Aging as a Social Process : Canadian Perspectives. Don Mills, ON: Oxford University Press (2008).

[49] Zwijsen SA Niemeijer AR Hertogh CMPM. Ethics of using assistive technology in the care for community-dwelling elderly people: an overview of the literature. Aging Ment Heal. (2011) 15:419–27. 10.1080/13607863.2010.543662 21500008

[50] Velaga NR Beecroft M Nelson JD Corsar D Edwards P. Transport poverty meets the digital divide: accessibility and connectivity in rural communities. J Transp Geogr. (2012) 21:102–12. 10.1016/j.jtrangeo.2011.12.005

[51] Woolrych R Sixsmith A Mortenson B Robinovitch S Feldman F. The nature and use of surveillance technologies in residential care. In: Bertino E Gao W Steffen B Woeginger G Yung M editors. Lecture Notes in Computer Science (including subseries Lecture Notes in Artificial Intelligence and Lecture Notes in Bioinformatics). Berlin: Springer (2013). p. 1–9.

[52] Righi V Sayago S Blat J. Urban ageing: technology, agency and community in smarter cities for older people. In: ACM's International Conference Proceedings Series. New York, NY (2015). p. 119–28.

[53] Lemlouma T Laborie S Roose P. Toward a context-aware and automatic evaluation of elderly dependency in smart homes and cities. In: 2013 IEEE 14th International Symposium on a World of Wireless, Mobile and Multimedia Networks, WoWMoM. Madrid (2013).

[54] Graham S Marvin S. Telecommunications and the City: Electronic Spaces, Urban Places (2002).

[55] Guo B Bricout JC Huang J. A common open space or a digital divide? A social model perspective on the online disability community in China. Disabil Soc. (2005) 20:49–66. 10.1080/0968759042000283638

[56] Woolrych R. Ageing and technology: creating environments to support an ageing society (keynote). Gerontechnology. (2016) 15:65–97. 10.4017/gt.2016.15.2.005.00

[57] Sixsmith A Carrillo M Phillips D Lansley P Woolrych R. International initiatives in technology and aging. In: Sixsmith A Gutman G editors. Technologies for Active Aging. Boston, MA: Springer US (2013). p. 201–21.

[58] Fang ML Canham SL Battersby L Sixsmith J Wada M Sixsmith A. Exploring privilege in the digital divide: implications for theory, policy, and practice. Gerontologist. (2019) 59:E1–15. 10.1093/geront/gny037 29750241

[59] Selwyn N. Apart from technology: understanding people's non-use of information and communication technologies in everyday life. Technol Soc. (2003) 25:99–116. 10.1016/S0160-791X(02)00062-3

[60] Dwyer P Hardill I. Promoting social inclusion? The impact of village services on the lives of older people living in rural England. Ageing Soc. (2011) 31:243–64. 10.1017/S0144686X1000085130886898

[61] Owuor J Larkan F Kayabu B Fitzgerald G Sheaf G Dinsmore J. Does assistive technology contribute to social inclusion for people with intellectual disability? A systematic review protocol. BMJ Open. (2017) 8:e017533. 10.1136/bmjopen-2017-017533 29440153 PMC5829787

[62] Krieg RM. Information technology and low-income, inner-city communities. J Urban Technol. (2007) 3:1–17. 10.1080/10630739508724513

[63] Wagner N Hassanein K. Computer use by older adults: a multi-disciplinary review. Comput Human Behav. (2010) 26:870–82. 10.1016/j.chb.2010.03.029

[64] Townsend L Sathiaseelan A Fairhurst G Wallace C. Enhanced broadband access as a solution to the social and economic problems of the rural digital divide. Local Econ. (2013) 28:580–95. 10.1177/0269094213496974

[65] Hodge H Carson D Newman L Garrett J. Using Internet technologies in rural communities to access services: the views of older people and service providers. J Rural Stud. (2017) 54:469–78. 10.1016/j.jrurstud.2016.06.016

[66] Bai Z Duan X Peng J Wu H Tan J. Urbanization of Chongqing Theory Practice. Ke xue chu ban she (2010). Available online at: https://baike.baidu.com/item/重庆市城镇化发展的理论与实践 (accessed March 28, 2019).

[67] Chongqing Municipal Bureau of Statistics.统计年鉴 - 重庆市统计局 (2020). Available online at: http://tjj.cq.gov.cn/zwgk_233/tjnj/ (accessed January 12, 2021).

[68] Caprotti F Liu D. Platform urbanism and the Chinese smart city: the co-production and territorialisation of Hangzhou City Brain. Geo J. (2020) 1–15. 10.1007/s10708-020-10320-2 33162645 PMC7607375

[69] Guo M Liu Y Yu H Hu B Sang Z. An overview of smart city in China. China Commun. (2016) 13:203–11. 10.1109/CC.2016.748998727295638

[70] DSJJ GOV. Notice on the Announcement of the 2019 Chongqing New Type of Smart City Construction Demonstration Project List (关于2019年重庆市新型智慧城市建设示范项目名单公示的通知). Chongqing: Chongqing Big Data Application and Development Administration (2019).

[71] Ministry of Housing and Urban-Rural Development. 国家智慧城市试点. Ministry of Housing and Urban-Rural Development (2013). Available online at: https://baike.baidu.com/item/国家智慧城市试点 (accessed October 28, 2018).

[72] Yuzhong Goverment. 人口状况_ 重庆市渝中区人民政府 (2021). Available online at: http://www.cqyz.gov.cn/zjyz/rkzk/202104/t20210415_9173750_wap.html (accessed September 9, 2021).

[73] Braun V Clarke V. Thematic analysis. In: Cooper H Camic PM Long DL Panter AT Rindskopf D Sher KJ editors. APA Handbook of Research Methods Psychology, Vol. 2, Research Designs: Quantitative, Qualitative, Neuropsychological, and Biological. Washington, DC: American Psychological Association (2012). p. 57–71.

[74] Kitchin R. Reframing, reimagining and remaking smart cities. In: Coletta C Evans L Heaphy L Kitchin R editors. Creating Smart Cities. London: Routledge (2018). p. 219–30.

[75] Kempin Reuter T. Human rights and the city: including marginalized communities in urban development and smart cities. J Hum Rights. (2019) 18:382–402. 10.1080/14754835.2019.1629887

[76] Norstrand JA Xu Q. Social capital and health outcomes among older adults in China: the urban-rural dimension. Gerontologist. (2012) 52:325–34. 10.1093/geront/gnr072 21746837

[77] Allam Z. Contextualising the smart city for sustainability and inclusivity. New Des Ideas. (2018) 2:124–7. Retrieved from: http://jomardpublishing.com/UploadFiles/Files/journals/NDI/V2N2/Zaheer%20Allam.pdf

[78] Huber L Watson C. Technology: education and training needs of older adults. Educ Gerontol. (2014) 40:16–25. 10.1080/03601277.2013.768064

[79] Deng Z Treiman DJ. The impact of the cultural revolution on trends in educational attainment in the People's Republic of China. Am J Sociol. (1997) 103:391–428. 10.1086/231212

[80] McCreadie C Tinker A. The acceptability of assistive technology to older people. Ageing Soc. (2005) 25:91–110. 10.1017/S0144686X0400248X16035974

[81] Rogers WA Mitzner TL. Envisioning the future for older adults: autonomy, health, well-being, and social connectedness with technology support. Futures. (2017) 87:133–9. 10.1016/j.futures.2016.07.002 28458395 PMC5407378

[82] Liu L Stroulia E Nikolaidis I Miguel-Cruz A Rios Rincon A. Smart homes and home health monitoring technologies for older adults: a systematic review. Int J Med Inform. (2016) 91:44–59. 10.1016/j.ijmedinf.2016.04.007 27185508

[83] Mortenson WB Sixsmith A Woolrych R. The power(s) of observation: theoretical perspectives on surveillance technologies and older people. Ageing Soc. (2015) 35:512–30. 10.1017/S0144686X13000846 29307944 PMC5756081

[84] Van Zoonen L. Privacy concerns in smart cities. Gov Inf Q. (2016) 33:472–80. 10.1016/j.giq.2016.06.004

[85] Givens JW Lam D. Smarter cities or bigger brother? How the race for smart cities could determine the future of china, democracy, and privacy. Fordham Urb LJ. (2020) 47:829–83. Retrieved from: https://ir.lawnet.fordham.edu/ulj/vol47/iss4/2/

[86] Atlam HF Wills GB. IoT security, privacy, safety and ethics. In: Fortino G Liotta A editors. Internet of Things. Cham: Springer International Publishing (2020). p. 123–49.

[87] Righetti F Vallati C Anastasi G. IoT applications in smart cities: a perspective into social and ethical issues. In: Proceedings - 2018 IEEE International Conference on Smart Computing, SMARTCOMP. Taormina (2018). p. 387–92.

[88] Kitchin R Dodge M. The (In)Security of smart cities: vulnerabilities, risks, mitigation, and prevention. J Urban Technol. (2019) 26:47–65. 10.1080/10630732.2017.1408002

[89] Belanche D Casaló LV Orús C. City attachment and use of urban services: benefits for smart cities. Cities. (2016) 50:75–81. 10.1016/j.cities.2015.08.016

[90] Liddle J Stuart A Worthy P Levine M Kastelle T Wiles J. “Building the threads of connection that we already have”: the nature of connections via technology for older people. Clin Gerontol. (2021) 44:406–17. 10.1080/07317115.2020.1852638 33263493

[91] Bhachu AS Hine N Arnott J. Technology devices for older adults to aid self management of chronic health conditions. In: ASSETS'08: The 10th International ACM SIGACCESS Conference on Computers and Accessibility. Halifax (2008). p. 59–66.

[92] Doyle J Caprani N Bond R. Older adults' attitudes to self-management of health and wellness through smart home data. In: Proceedings of the 2015 9th International Conference on Pervasive Computing Technologies for Healthcare, Pervasive Health. Istanbul: Institute of Electrical and Electronics Engineers Inc. (2015). p. 129–36.

[93] Meystre S. The current state of telemonitoring: a comment on the literature. Telemed J e-Health. (2005) 11:63–9. 10.1089/tmj.2005.11.63 15785222

[94] Kitchin R. Making sense of smart cities: addressing present shortcomings. Cambridge J Reg Econ Soc. (2015) 8:131–6. 10.1093/cjres/rsu02715366237

[95] Oswald F Hieber A Wahl HW Mollenkopf H. Ageing and person-environment fit in different urban neighbourhoods. Eur J Ageing. (2005) 2:88–97. 10.1007/s10433-005-0026-5 28794721 PMC5547678

[96] Phillips J Walford N Hockey A. How do unfamiliar environments convey meaning to older people? Urban dimensions of placelessness and attachment. Int J Ageing Later Life. (2011) 6:73–102. 10.3384/ijal.1652-8670.116273

[97] Sixsmith A. Technology and the challenge of aging. In: Technologies for Active Aging. Boston, MA: Springer (2013). p. 7–25.

[98] Kitchin R Cardullo P Di Feliciantonio C. Citizenship, justice, and the right to the smart city. In: Cardullo P Di Feliciantonio C Kitchin R editors. The Right to the Smart City. Bingley: Emerald Publishing Limited (2019). p. 1–24.

[99] Lee MA Ferraro KF Kim G. Digital technology use and depressive symptoms among older adults in Korea: beneficial for those who have fewer social interactions? Aging Ment Heal. (2020) 25:1839–47. 10.1080/13607863.2020.1839863 33131296

